# Potent Anti-Tumor Effect Generated by a Novel Human Papillomavirus (HPV) Antagonist Peptide Reactivating the pRb/E2F Pathway

**DOI:** 10.1371/journal.pone.0017734

**Published:** 2011-03-15

**Authors:** Cai-ping Guo, Ke-wei Liu, Hai-bo Luo, Hong-bo Chen, Yi Zheng, Shen-nan Sun, Qian Zhang, Laiqiang Huang

**Affiliations:** 1 School of Life Sciences, Tsinghua University, Beijing, People's Republic of China; 2 The Shenzhen Key Lab of Gene and Antibody Therapy, Center for Biotech & BioMedicine and Division of Life Sciences, Graduate School at Shenzhen, Tsinghua University, Shenzhen, Guangdong, People's Republic of China; Florida International University, United States of America

## Abstract

Human papillomavirus type 16 (HPV16) E7 is a viral oncoprotein believed to play a major role in cervical cancer. In this study, an antagonist peptide against HPV16E7 protein was first identified from screening the c7c phage display peptide library. The binding specificity and affinity of the selected peptide to HPV16E7 were tested by competitive enzyme-linked immunosorbent assay (ELISA). The antagonist peptide showed obvious anti-tumor efficacy both in cell lines and animal tumor models. Significant cell proliferation inhibition with high specificity was noted when HPV16-positive cells were treated with the peptide. This anti-tumor efficacy was resulted from overriding the activities of HPV16E7 and reactivating the pRb/E2F pathway, as shown by a series of experiments. Flow cytometry analysis revealed that the selected peptide induced G1 arrest in a dose-dependent manner. Competitive ELISA, pull down, and Co-IP experiments indicated that the selected peptide disrupted the interaction between HPV16E7 and pRb proteins both *in vitro* and *in vivo*. Luciferase reporter assay verified that transcription activities of E2F were suppressed by the peptide through restoration of pRb. RT-PCR and Western blot revealed that it reduced cyclins A, D1, and E1 expression, and led to HPV16E7 protein degradation, but pRb protein stabilization. The current study suggests that this specific peptide may serve as a potential therapeutic agent for HPV16-positive cervical cancer.

## Introduction

Cervical cancer is the second leading cause of cancer death in women worldwide. In the past 20 years, a large number of studies have suggested that the role of high-risk human papillomavirus (HPV) is critical for the occurrence and development of cervical cancer [1]. Several reports stated that HPV was detected in 99.7% of cervical tumor specimens, 50–60% of which were HPV 16-positive [2], [3], [4]. HPV E6 and E7 have been proven to play important roles in malignant transformation. They are expressed in HPV-transduced cells to inactivate tumor suppressor proteins such as p53 and pRb, respectively, leading to cell cycle disorder, telomerase activation, and cell immortalization. Their continued expression is essential for the malignant transformation and maintenance of tumor cells [5], [6], [7], [8], [9].

High-risk HPV E7 associates with the retinoblastoma family of proteins (pRb, p107 and p130), induces their ubiquitin-mediated proteolysis, and disrupts their association with the E2F family of transcription factors, subsequently transactivating cellular proteins required for DNA replication. Moreover, E7 binds and induces the proteasome-mediated degradation of pRb, p107 and p130 proteins [10], [11], [12], [13], [14], [15], [16]. Additional E7-interacting proteins have been found in recent years, including transcription factors p300, CBP and pCAF [17], [18]. More recently, E7 has been shown to interact with a pRb-associated protein, p600 [19], [20] and induce genetic instability, predominantly via its ability to induce abnormal centrosome numbers and multipolar mitotic spindles [21].

As these viral proteins are not expressed in normal cells, E7 protein has become an important research target for HPV therapy. Strategies for achieving specific and selective abrogation of E7 protein have been proposed as a rational therapeutic approach for HPV-positive carcinoma of the cervix [22]. A few studies have shown that antisense and peptide aptamers targeting HPV E6/E7 can induce target cell apoptosis [23], [24], [25]. Therefore, finding inhibitory peptides that target E7 and rescue the tumor-suppressing activity of pRb may be one of the notable means of therapy for HPV-positive cervical carcinoma.

In this study, we screened for HPV16E7 antagonists from a C7C phage library, and a new peptide, which specifically binds to the HPV16E7 protein, was selected. The peptide repressed the proliferation of HPV16-positive cervical carcinoma cells selectively. The proliferation inhibition was associated with cell cycle arrest and function restoration of pRb, which suppresses E2F transactivation and down-regulates a group of cell-cycle progressing-related genes, including cyclins A, D and E. More encouragingly, subcutaneous tumor model experiments demonstrated that the peptide could suppress the tumor xenografts growth significantly. In light of these results, there is a strong evidence to consider this selected peptide a “good candidate” for the treatment of HPV16-positive cancer.

## Results

### Phage Display Screening and Peptides Synthesis

The full-length HPV16E7 protein was expressed in E. *coli* BL21 and purified by immobilized-metal affinity chromatography on Ni-NTA agarose beads as instructed by the manufacturer and was used as a target for the panning of a random heptapeptide phage display library. After three rounds of bio-panning, nearly 1×10^3^ enrichments of positive-binding clones were yielded. The isolated phage clones' ability to bind to the HPV16 E7 protein was tested by ELISA. Seven of the 40 isolated clones showed high absorbance values in the ELISA assay (>3×absorbance values of control). They were identified as positive phage clones (data not shown).

The nucleotide sequences coding for peptides on the positive phage clones were determined by Shanghai Sangon Biological Engineering Technology and Service Co., Ltd. (Shanghai, P.R. China). Results showed that all eight clones we obtained from the screening had the same sequence PKGLDWC. In data bank searches, we did not find any obvious similar sequences in naturally occurring E7 binding proteins, including pRb, or human proteins. The corresponding peptide SACPKGLDWCGG (C&C bridged by disulfide bonds), without or with 5-FAM labeling, were chemically synthesized for further study (named “Pep-7” and “F-pep,” respectively). The peptide with the same amount, but random sequence, of amino acids was synthesized as negative control peptide (named “N-pep”).

### Both the Positive Clones and Selected Peptide Pep-7 Have Specific Affinity to HPV16E7

**Figure 1:**
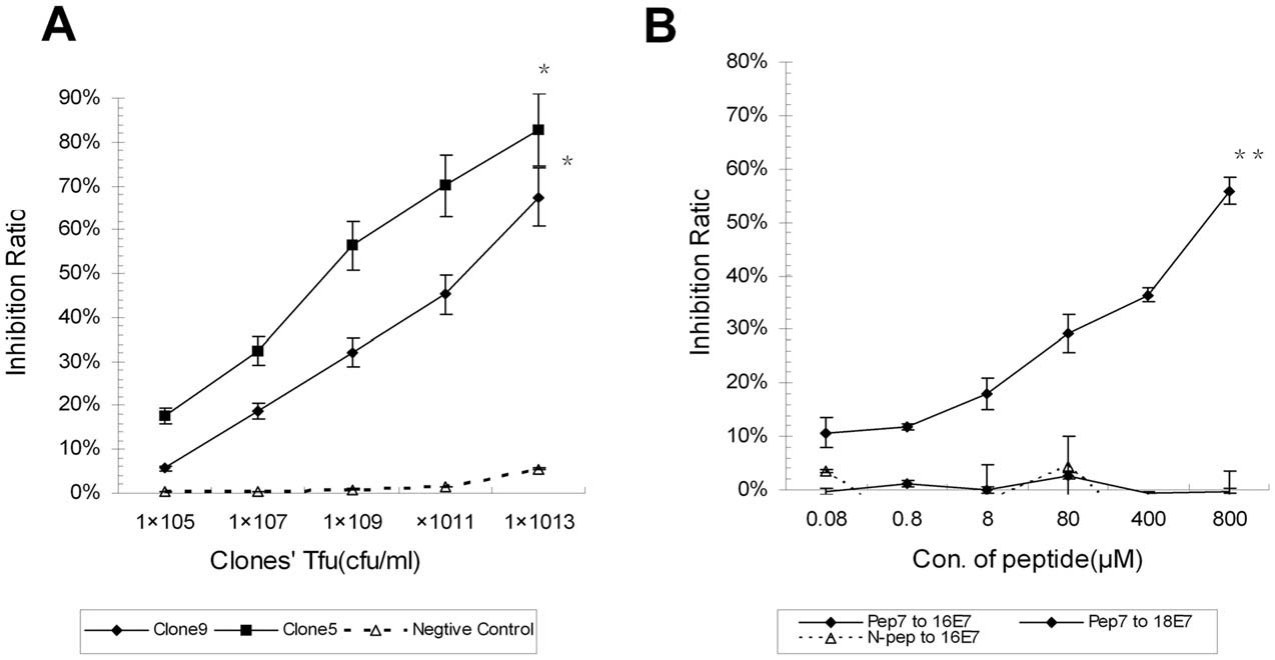
Competitive ELISA Assay. (A) Positive clones' affinities to 16E7 were tested by competitive ELISA. HPV16 E7 protein was coated in microtiter wells. Positive phage Clones 5 and 9 and negative control phage at concentrations of 10^13^ cfu/ml to 10^5^ cfu/ml, competed with HPV16E7 mAb, respectively, for binding the coated E7 protein. (B) The synthetic peptides were tested for affinities to 16E7. Pep-7 or N-pep at concentrations of 0.08 µM to 800 µM competed with HPV16 E7 mAb and HPV18 E7 anti-serum for binding to target. Data were presented as the mean ± SE from three independent experiments (^*^, p<0.02; ^**^, p<0.01).

The specificity affinities of the positive clones and Pep-7 were determined by competitive ELISA. Positive clone B-5 and B-9 and library phages (negative control) at concentrations of 10^13^ CFU/ml to 10^5^ CFU/ml were mixed with HPV16E7 mAb, and then incubated with the coated E7 protein. HPV16E7 mAb alone, incubated with the coated E7 protein, gave the benchmark absorption value. Absorbance values and inhibitory ratio were collected and calculated as outlined in Materials and Methods. Both clones B-5 and B-9 were revealed to have high affinities for binding to the HPV16 E7 protein. The binding inhibitory ratio of both clones at the concentration of 10^13^ CFU/ml were higher than 50%, while the negative control phage had a very low inhibitory rate (<5%, Figure 1A).

Similarly, Pep-7 and N-pep (as negative controls) at concentrations of 0.08 µM to 800 µM were used to compete with HPV16 E7 or HPV18 E7 antibodies for binding to their antigens. As shown in Figure 1B, the Pep-7 bound to HPV16 E7 with high affinity compared with the control peptide N-pep but did not bind to HPV18 E7. The differences between the binding affinities of Pep-7 to HPV16E7 and HPV18E7 were statistically significant (*p* = 0.004). Likewise, differences between the binding affinities of Pep-7 and N-pep to HPV16E7 were statistically significant (*p* = 0.015). Results revealed that the synthesized peptide Pep-7 had similar characteristics with the positive phage clones, binding to HPV16 E7 specifically.

### Peptide Internalization and Distribution

**Figure 2:**
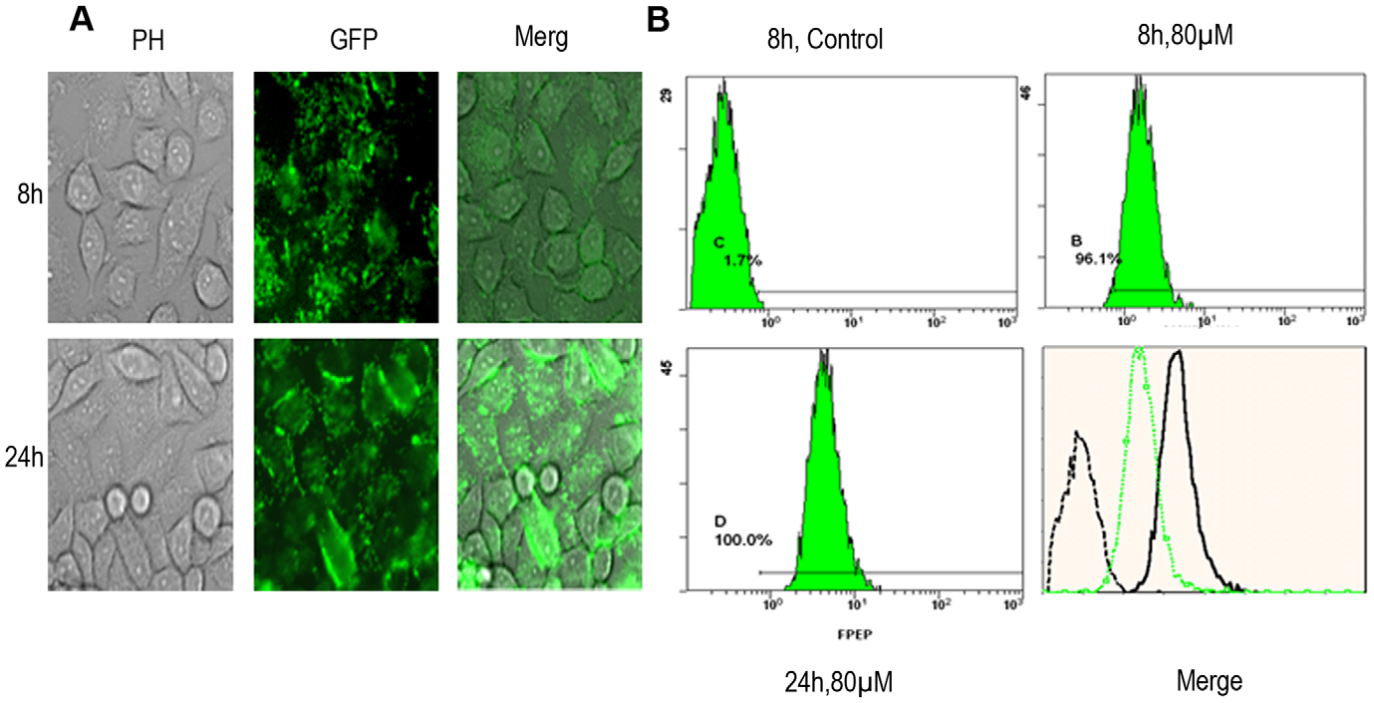
Cell-uptaking and Intracellular Location of the Selected Peptide. SiHa cells were observed under a fluorescence microscope (A) (scale bar: 50 µm) or analyzed by FCS (B) after 8 h or 24 h of incubation with 80 µM green fluorescent labeled F-pep.

The green fluorescent labeled “F-pep” was used to monitor cell uptake and intracellular location of the peptide. As shown in Figure 2B, when incubated with SiHa cells at 37°C, the peptide was uptaken by more than 96% cells after 8 h. Almost SiHa cells uptook the peptide after 24 h incubation. Visualization under fluorescence microscope indicated that F-pep was distributed in both cytoplasm and nucleus, which coincides with the subcellular location of HPV16 E7 protein (see Figure 2A) [26]. In contrast, cells incubated with equimolar 5-Fam did not show fluorescence. These results suggested that the peptide had strong cell-penetrating power, and it bound to E7 in SiHa cells.

### The Peptide Pep-7 Suppresses Cell Proliferation in HPV16 Positive Cell Lines

**Figure 3:**
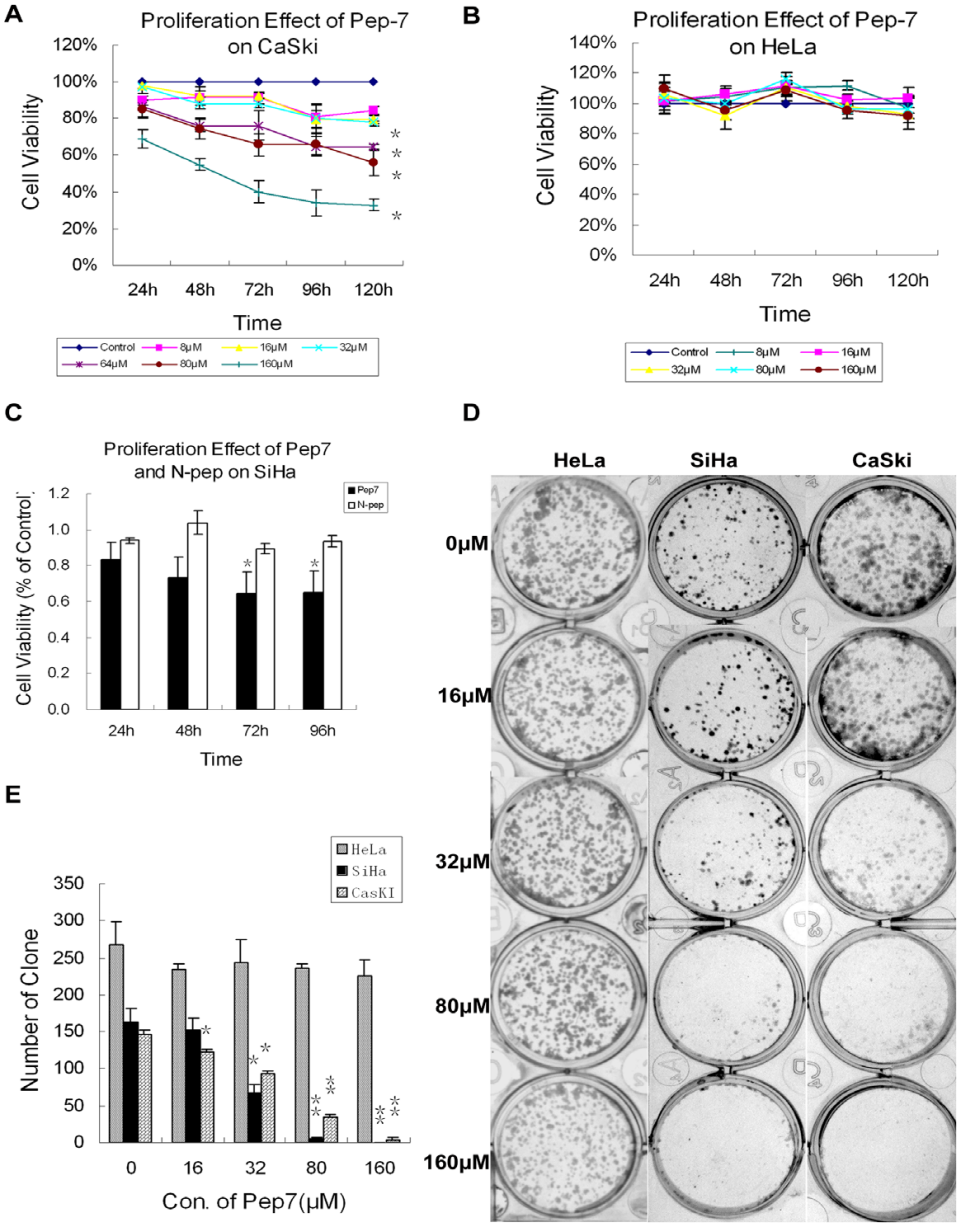
Effect of Pep-7 on Cell Proliferation. The effect of Pep-7 on cell growth at concentrations indicated were tested by MTT and clonogenic assay. Cell viability was presented as a percentage of A540 relative to the control cells. MTT experiments (A to C) results are presented as mean ± SE of data obtained from at least two independent run in sextuplicate. (A) Pep-7 effect on CaSki cell growth; (B) Pep-7 effect on HeLa cell growth; (C) comparing effect of 80 µM N-pep and Pep-7 on SiHa cell growth; (D) Photograph of cell clones stained with crystal violet; and (E) The average ± SE clone numbers of each group from three independent experiments (^*^, p<0.02; ^**^, p<0.01).

To determine whether the peptide could interfere with the growth of HPV16-positive mammalian cells, we first tested the effect of Pep-7 on the proliferation of HPV16-positive cervical carcinoma cells CaSki, SiHa and the HPV18-positive cervical cancer cell line HeLa by MTT at different time points, as indicated in Figure 3. The proliferation of HPV16-positive cells was repressed significantly by Pep-7 in a dose- and time-dependent manner (Figure 3A), but not by the negative control peptide N-pep (Figure 3C). On the other hand, Pep-7 could not repress the proliferation of HeLa cells (Figure 3B). The effect of Pep-7 on the proliferation of other HPV-negative cell lines including CNE, HaCat, Cos7, and NIH 3T3, was also tested. Again, Pep-7 showed negligible effect on these cells (data not shown).

The specific growth suppression of Pep-7 was further assessed by colony formation experiments. In this study, SiHa, CaSki and HeLa cells were treated with or without Pep-7 at concentrations indicated in Figure 3. However, only the growth of SiHa and CaSki cells was inhibited by Pep-7. The differences in colony number between the control (0 µM group) and the Pep-7-treated group were significant (*p*<0.02) when the concentrations of Pep-7 increased up to 32 µM. The colony formation of HeLa cell was not influenced by Pep-7 at concentrations of 16 through 160 µM (*p*>0.1).

### Pep-7 Induces Cell Cycle Arrest in Parallel with Modulation of mRNA and Protein Levels

**Figure 4:**
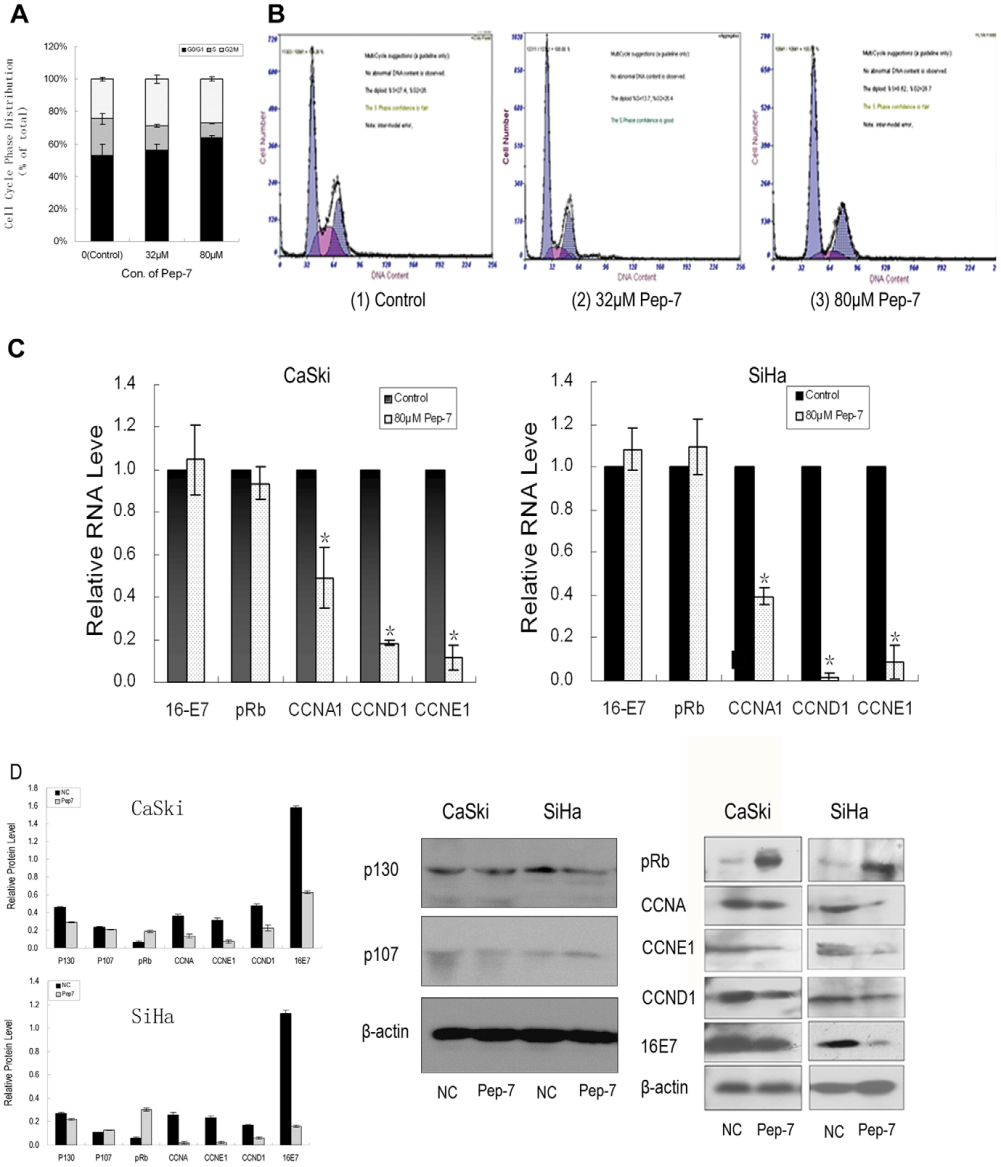
Pep-7-Induced Cell Cycle Arrest and Relative Gene Expression. (A) Statistical data of cell phase distribution from three experiments; (B) Representative images of flow cytometry, with (1) showing the results for the control, (2) showing the results for the 40 µM Pep-7 treatment, and (3), for the 80 µM Pep-7 treatment; (C) The RNA level of pRb, HPV16E7, and cyclins A, D1, and E1 in SiHa and CaSki cells treated with or without 80 µM Pep-7; (D) The protein level of p107, p130 and the above genes in SiHa and CaSki cells treated without(Lane NC) or with 80 µM Pep-7 (Lane Pep-7). β-actin was used as loading control, and protein level was calculated from the band intensities of each proteins ralative to that of β-actin. Results represent means ± SE (^*^, p<0.02).

In this study, the cell cycle analysis revealed that the cell cycle arrest induced by Pep-7 contributed much to the suppression of cell proliferation. SiHa cells were treated with or without Pep-7 and then subjected to PI staining cell cycle analysis by flow cytometry as described in Materials and Methods. As shown in Figure 4 (A and B), Pep-7 inhibited G1-to-S phase transition of SiHa cells significantly (*p*<0.02). The percentage of cells in S-phase dropped from 25.96% (control) to 14.75% when treated with 32 µM Pep-7, and to 8.75% when treated with 80 µM Pep-7.

To address the exact mechanism of Pep-7-induced G1 arrest, the expression of different proteins involved in regulating G1-to-S transition was monitored by qRT-PCR and Western blot. Data showed that treatment of SiHa and CaSki cells with 80 µM Pep-7 significantly reduced the expressions of Cyclins A, D1 and E1 at both RNA and protein levels. On the other hand, pRb protein level was increased but HPV16E7 protein was reduced by Pep-7, though RNA levels of these genes were not affected by Pep-7 [Figure 4 (C and D)]. This suggests that the peptide may modulate transcription of the cyclins while affecting the stability of HPV16E7 and pRb protein.

### Pep-7 Affects Stabilization but Not Phosphorylation of pRb

**Figure 5:**
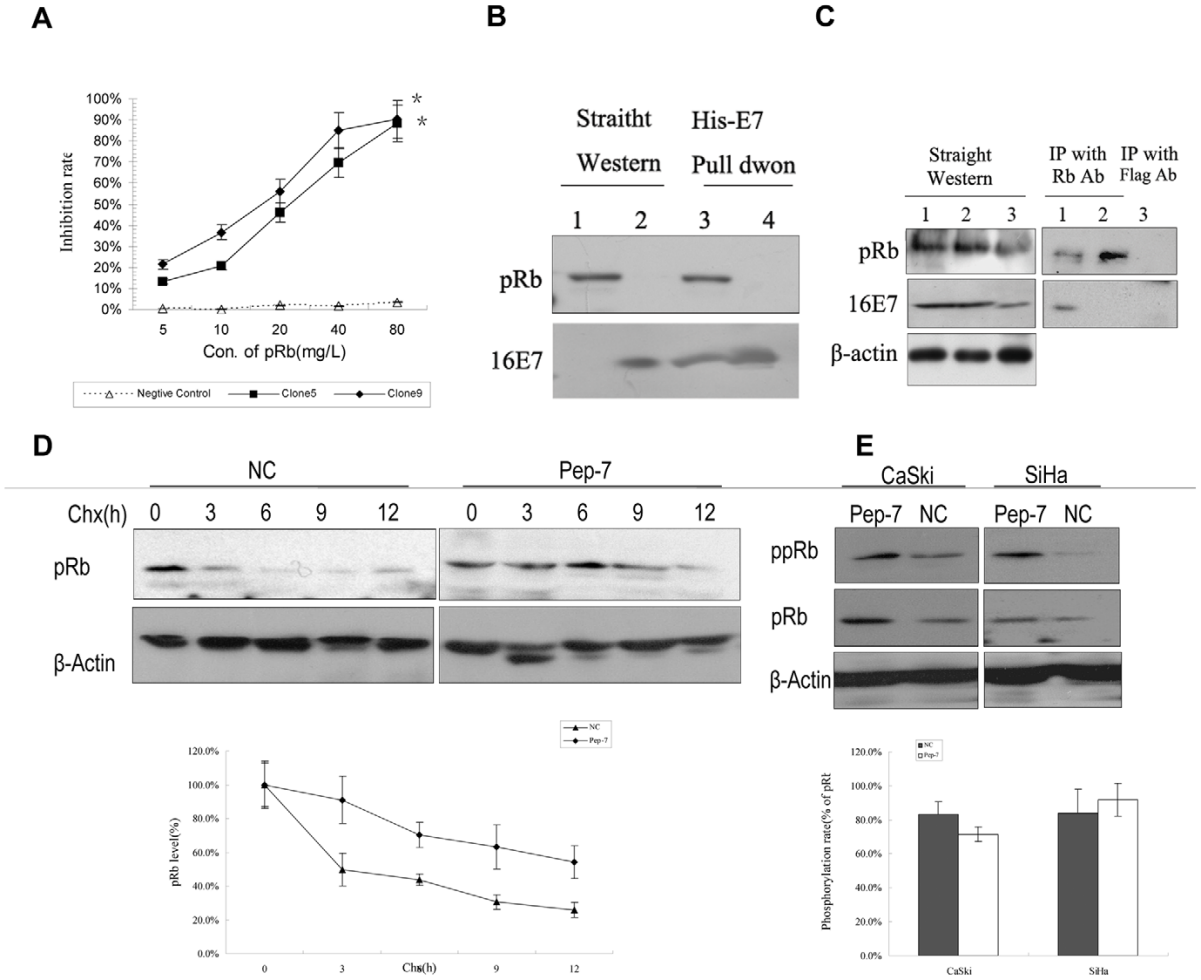
Pep-7 Blocks Interaction Between pRb and HPV16E7 and Stabilizes pRb. (A) Competition between positive clones and pRb at concentrations indicated for binding to HPV16 E7. (B) His-pull down experiment. Lane 1, 293T cell lysates tested by Western blot directly; Lane 2, HPV16 E7 protein tested by Western blot directly; Lane 3, pRb pulled down by HPV16 E7-His-band resin from 293T cell lysates; Lane 4, pRb pulled down by HPV16 E7-His-band resin from 293T cell lysates in the presence of Pep-7. (C), Co-IP of HPV16 E7 and pRb. Cos7 cells co-transfected with His-pRb or YFP-HPV16E7 were treated with (lane 2) or without Pep-7 (lane 1 and 3). The cell lysates (left panel) or immunoprecipitates of pRb antibody (right panel, lane 1 and 2) or Flag antibody (right panel, lane 3) were blotted with HPV16 E7 or pRb antibodies; (D) half-life study of pRb with or without Pep-7 treatment. SiHa cells incubated with or without 80 µM Pep-7 were treated with 50 µg/ml cycloheximide for 12 h, and the cells were harvested for Western blotting at time points indicated; (E) phosphorylation of pRb assay in cells treated with or without Pep-7. SiHa cells were harvested and subjected to Western blotting probed with pRb and Phospho-Rb antibodies respectively, post 24 h incubation with or without 80 µM Pep-7. β-actin was used as loading control, and protein level was calculated from the band intensities of each proteins relative to that of β-actin (^*^, p<0.02; ^**^, p<0.01).

Measurements of pRb half-life were carried out. SiHa cells were treated with or without Pep-7 for 24 h after seeded in 6-well plates overnight, and then cycloheximides were added at a concentration of 50 µg/ml, and cells were collected at different time points for Western blot assay. A summary of three experiments is shown in Figure 5D. pRb half-life was significantly expanded in Pep-7 treated groups, compared with control, supporting that Pep-7 induced pRb stabilization. In contrast, the phosphorylation of pRb was not affected significantly by Pep-7 as shown in Figure 5E.

### Pep-7 Blocks Interaction Between pRb and HPV16E7 and Reactivates pRb/E2F Pathway

The competitive ELISA test between positive clones and pRb showed that the binding between positive clones and HPV16E7 could be disrupted by the pRb protein (Figure 5A). The inhibition ratio of binding of clones B-5 and B-9 with HPV16E7 was increased with an increase in pRb concentration. The results suggest that the selected peptide might compete with pRb for binding to HPV16E7.

To determine whether the peptide disrupts the binding of HPV16E7 with pRb, the Pep-7 effect on the interaction between pRb and HPV16E7 was evaluated. First, we performed His-pull down assay. Results showed that His-HPV16E7 fusion protein could not pull down pRb from 293T cell lysates with Pep-7 present. Without Pep-7 interference, however, pRb was pulled down by His-HPV16E7 fusion protein (Figure 5B). In addition, the Co-IP assay performed using Cos7 cells co-transfected with HPV16E7 and pRb constructs yielded results similar to those of the pull-down test. HPV16E7 could be immunoprecipitated by the pRb antibodies without the presence of Pep-7, but when cells were treated with Pep-7, HPV16E7 could not be immunoprecipitated by the pRb antibodies (Figure 5C). These results indicated that Pep-7 disrupted the binding between the HPV16E7 and pRb proteins both *in vitro* and *in vivo*.

**Figure 6:**
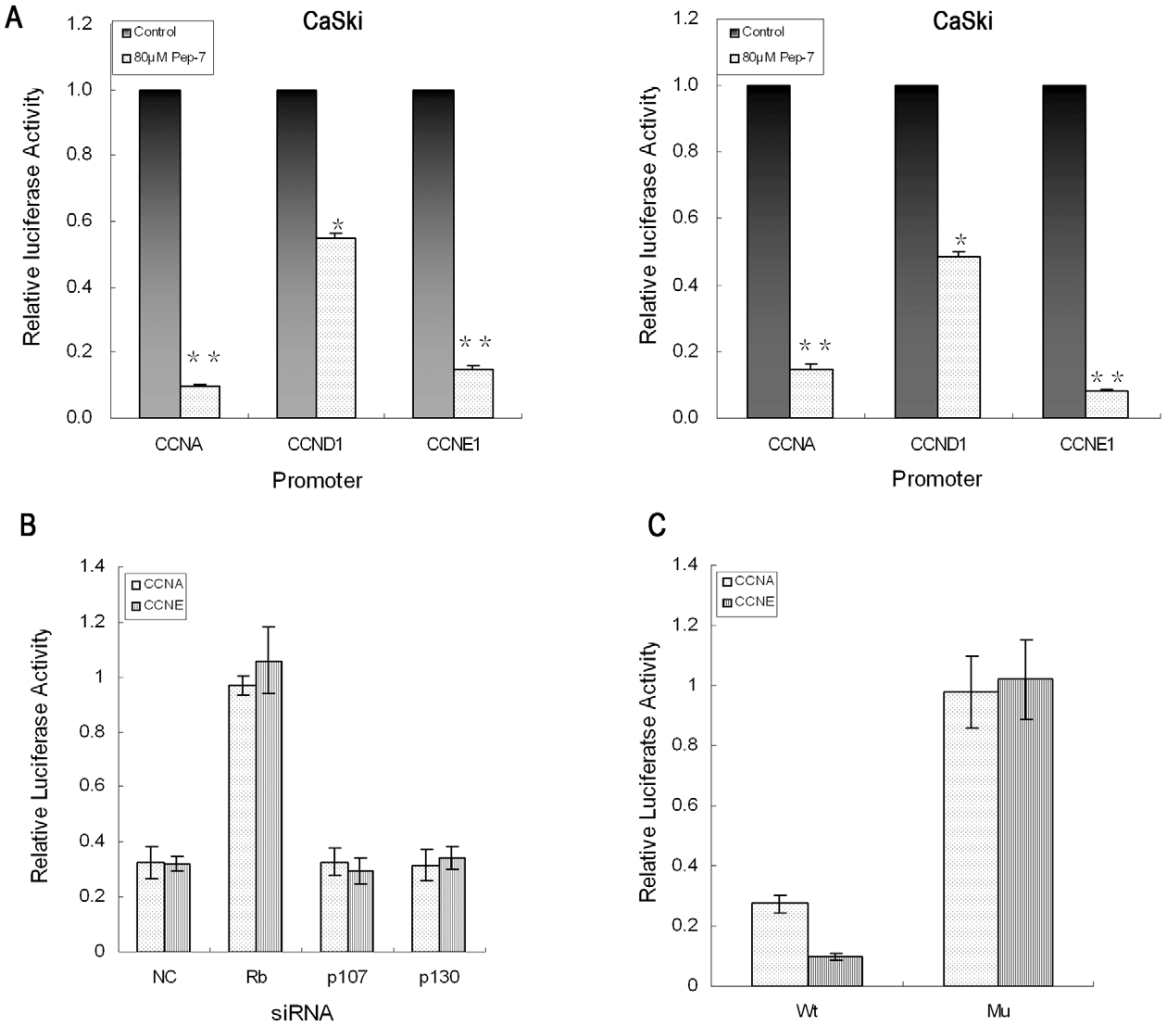
Luciferase Reporter Assay. The luciferase reporter activity of each sample was normalized against the corresponding protein content prior to calculation of the fold induction value relative to the controls. (A) Luciferase activity of E2F-dependent-promoters; (B) Comparison of the luciferase activity of E2F-dependent promoters with pRb, p107, p130, or negative control siRNA treatment; (C) Luciferase activity of mutant promoters (E2F binding sites mutated). Results represent means ± SE from three experiments (^*^, p<0.02; ^**^, p<0.01).

As pRb represses the transcription factor E2F, which regulates the transcription of genes such as cyclin E, its release by Pep-7 from E7 may restore the down-regulation of transcriptional activity of E2F. The speculation was verified by E2F-responsive reporter assays. Reporter constructs with cyclin A, E1 and D1 promoters containing E2F binding sites were transfected into SiHa and CaSki cells. The cells were treated with Pep-7 24 h post transfection and then subjected to luciferase assay. As shown in Figure 6A, Pep-7 resulted in remarkable down-regulation of activities of cyclin A, E1 and D1 promoters. On the other hand, when the E2F binding sites in cyclin A and E1 promoters were mutated, the down-regulations of the promoters by Pep-7 were abolished. Figure 6C shows that the activities of mutated promoter constructs, pMCCNA-Luc and pMCCNE1-Luc in SiHa cells treated with Pep-7 have negligible differences from those in control groups.

In order to further determine the involvement of pRb-E2F pathway in Pep-7 function, members of Rb family including pRb, p130 and p107 were knocked-down by siRNA to evaluate the effect of their deficiency on Pep-7 function. SiHa cells were co-transfected with E2F-responsive luciferase reporter plasmids and siRNA of Rb, p130 and p107, respectively. The cells were then treated with or without Pep-7 at 24 h post transfection for another 24 h before subjected to luciferase activities evaluation. Results in Figure 6B indicated that inhibition of pRb also abrogated the suppression of cyclin A, E1 and D1 promoter activities by Pep-7, whereas knocking down p130 or p107 did not affect the influence of Pep-7 on these promoters. This confirmed that pRb but not p130 or p107, was involved in activity repression of E2F by Pep-7.

### Pep-7 Inhibits Tumor Growth in Animal Model

**Figure 7:**
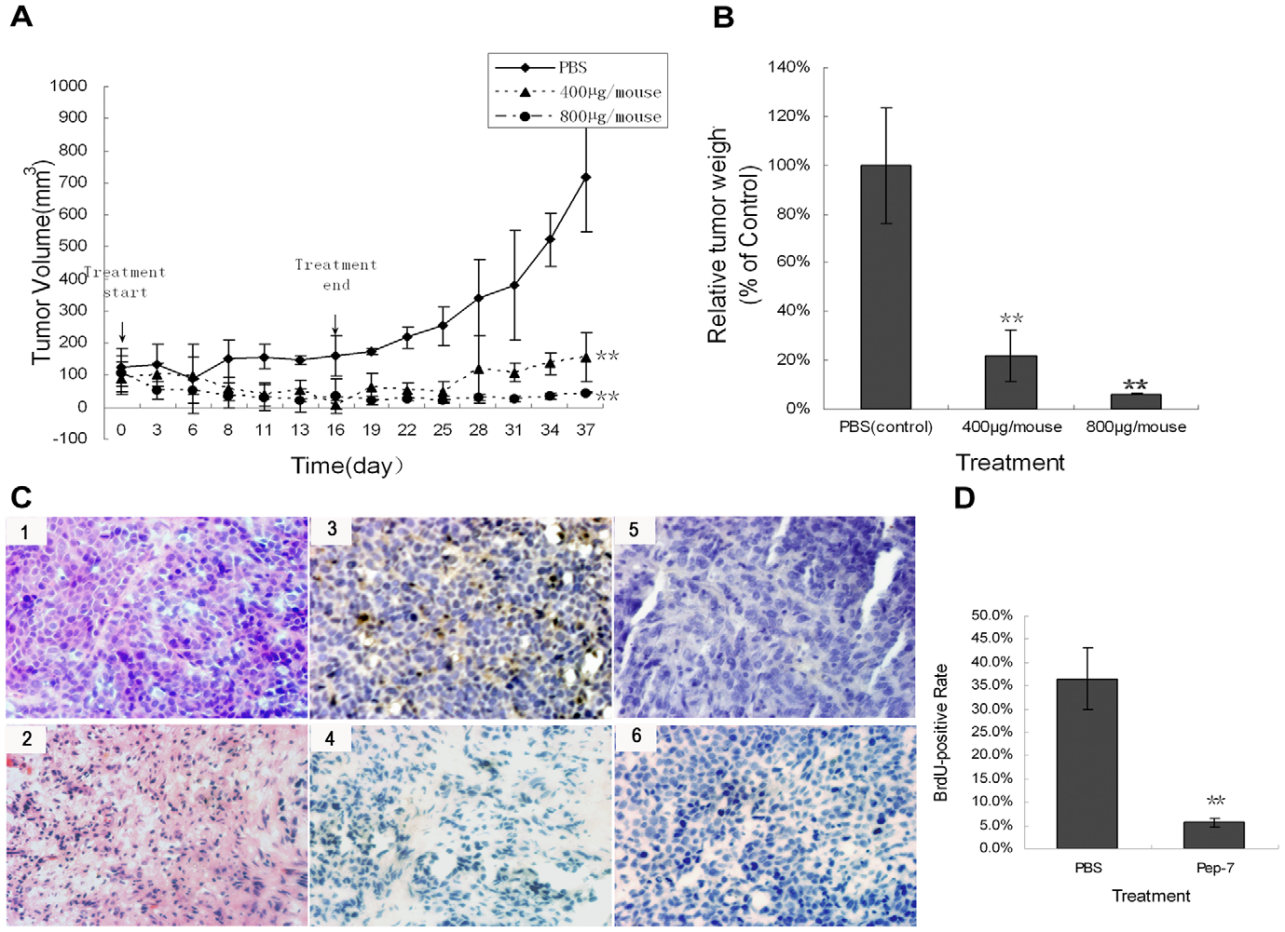
The anti-tumor efficacy of Pep-7 *in vivo*. Mice bearing subcutaneous tumors were treated for 16 days with intravenous injections of PBS (control) or Pep-7 at doses indicated every other days. Results represent means ± SE. (A) Tumor growth curve. Tumor volume was calculated using the formula “volume = length×width^2^/2,” (B) Tumor growth inhibition ratio, represented as relative tumor weight to control. Relative tumor weight (% of control) = average tumor weight of each dose treatment group/average tumor weight of control group”; (C) H/E staining and BrdU-incorporation staining of tumor tissues. (1) H/E staining of section from PBS treatment group; (2) H/E staining of tumor tissue from 400 µg Pep-7 treatment group; (3) BrdU antibody staining of section from mouse up-taking BrdU in PBS treatment group; (4) BrdU antibody staining of section from mouse up-taking BrdU in 400 µg Pep-7 treatment group; (5) BrdU antibody staining of section from mouse without up-taking BrdU in PBS treatment group (6) PBS staining of section from mouse up-taking BrdU in PBS treatment group; (D) statistical data of the BrdU-positive cells by accounting for 1000 total cells in each case (^*^, p<0.02; ^**^, p<0.01; Original magnification, 200×).

**Figure 8:**
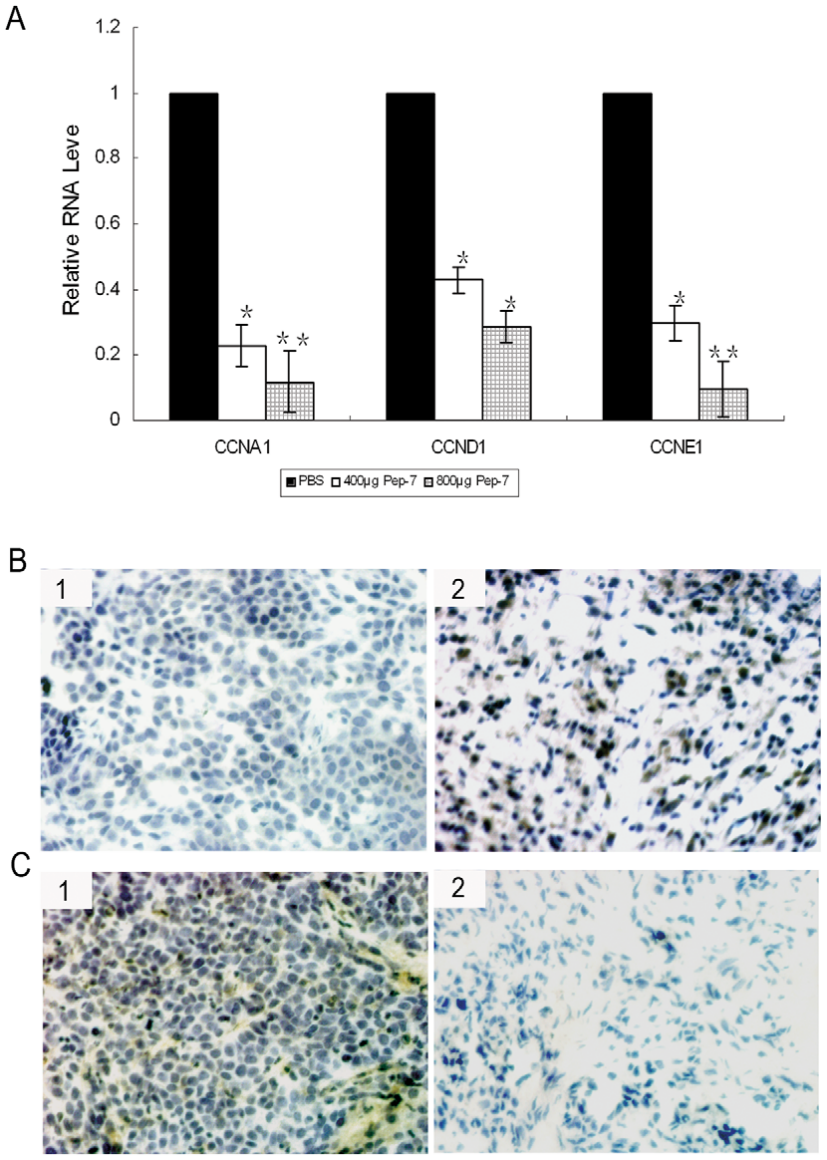
Gene Expression in Tumor Tissues. (A) Relative RNA levels of tumor tissues from qRT-PCR. (B) Immunohistochemical staining of pRB expression. panel 1, tumor tissue from control group; panel 2, tumor tissue from 400 µg Pep-7 treatment group; (C) Immunohistochemical staining of HPV16 E7 expression. panel 1, tumor tissue from control group; panel 2, tumor tissue from 400 µg Pep-7 treatment group (^*^, p<0.02; ^**^, p<0.01; Original magnification, 200×).

The anti-tumor efficacy of the selected peptide was further investigated *in vivo*; a subcutaneous tumor was constructed in nude mice by inoculation with 1.5×10^7^ SiHa cells. The mice were then treated for 16 days with intravenous injections of PBS or Pep-7 at doses of 400 µg/mouse and 800 µg/mouse, respectively. In the experiment, Pep-7 therapy showed significant anti-tumor efficacy (p<0.01, Figure 7). At the end of the experiment, the tumor growth was inhibited by 77.92% in 400 µg Pep-7/mouse dose group and 93.86% in 800 µg Pep-7/mouse dose group, respectively, compared with that in the control (PBS) group (Figure 7B). The H/E staining and BrdU uptake test of the tumor tissues also revealed that Pep-7 repressed tumor growth (Figure 7, C and D). In addition, immunohistochemical results showed that pRb protein level increased but HPV16E7 decreased in the Pep-7-treated group, compared with results of control group (Figure 8, B and C). Tissues from the 800 µg dose group were not examined due to the fact that the tumors were too small for the preparation of sections. Furthermore, qRT-PCR indicated that Pep-7 also reduced cyclins A, D1, and E1 expression at the RNA levels (Figure 8A). The in vivo results were consistent with the results in cell experiments, indicating that the Pep-7 inhibits tumor growth in animals through the same mechanism as in HPV-positive cells.

## Discussion

Current therapeutic strategies for HPV-associated tumors include surgical removal of the lesion, radiotherapy plus cisplatin-based chemotherapy, and non-specific stimulation of innate immunity. But the most commonly used cytotoxic therapy, cisplatin-based combination chemotherapy, has produced response rates ranging from 20% to 30% and overall survival of less than 10 months [27], [28], [29], [30]. Recently developed prophylactic HPV vaccines hold great promise for the prevention of HPV-associated cancers in the future [31]. However, prophylactic HPV vaccines do not have therapeutic effects against pre-existing HPV infections and HPV-associated lesions. Furthermore, due to the considerable burden of HPV infections worldwide, and the limitation of prophylactic HPV vaccine application by high costs and medical infrastructure problems [32], it would take decades for preventive vaccines to lower the prevalence of cervical cancer. In fact, the incidence of cervical cancer is estimated to increase by over 40% by the year 2020 [33]. Thus, novel treatment strategies are urgently required.

With the aim of obtaining reagents that could be utilized in HPV-associated tumor therapy, an antagonist peptide against HPV16E7 was isolated from a phage display library. Competitive ELISA assay revealed that the selected peptide Pep-7 bound to HPV16E7 protein with high specificity and affinity *in vitro*. This specific binding was peptide sequence-dependent because the negative peptide N-pep showed no affinity for HPV16E7 in the same experiment.

The effects on cellular proliferation of Pep-7 obtained using the MTT assay indicated that it significantly inhibited the viability of HPV16-positive cervical cancer cells. This inhibition was specific and dose-dependent. In fact, no significant anti-proliferative effect was observed either in HPV16-positive cells treated with the negative control peptide N-pep, or in HPV16-negative cells treated with Pep-7 [Figure 3 (A–C)]. As shown in Figure 3 (D and E), this specific inhibition was further confirmed by the colony formation assay, which indicated significant, dose-dependent Pep-7-blocked clone formation in SiHa and CaSki cells, but not in HeLa cells. This was in line with the competitive ELISA results, which showed that Pep-7 bound to HPV16E7 but not to HPV18E7 protein. Therefore, the inhibition of cell proliferation observed in SiHa cells and CaSki cells may be ascribed to HPV16E7 impairment by Pep-7. Moreover, the anti-tumor efficacy of Pep-7 was further determined in mammals. The therapeutic results were encouraging. The selected peptide showed significant anti-tumor efficacy on SiHa tumor xenografts (Figure 7), implying a potent anti-tumor perspective *in vivo* for HPV16-related neoplasms.

Suppression of cell proliferation may be the result of cell cycle arrest or apoptosis. It is well verified that the main function of HPV16E7 is inactivating retinoblastoma family of proteins pRb [22], [24] and leading to cell cycle disorder. Therefore, we focused our investigation on Pep-7's effect on cell cycle in the current study. Previous studies have indicated that HPV E7 protein prevents G1 arrest and drives cells from the G1 to the S phase in the cell cycle [34], [35]. In our study, the flow cytometry assay indicated that Pep-7 induced G1 arrest in SiHa cells [Figure 4 (A and B)], consistent with the cell proliferation inhibition results, arguing for that Pep-7 induces G1 arrest by eliminating the functions of HPV16E7. In addition, competitive ELISA assay showed that the selected phage clones competed with pRb for binding to HPV16E7 (Figure 5A). Pull down experiments and Co-IP tests also revealed that the selected peptide disrupted the association between HPV16E7 and pRb proteins [Figure 5 (B and C)]. It is reported that one mechanism of the inactivation of pRb by E7 is the targeted destabilization of pRb [11], [12], [36], [37], [38]. In agreement with these reports, our results demonstrated that Pep-7 could reduce the E7 protein level while inducing pRb protein augmentation in HPV16-positive cells. In contrast, Pep-7 did not exhibit an effect on the protein levels of other two members of pRb family, p107 and p130 (Figure 4). One explanation for this may be that E7 differentially regulates pRb, p130 and p107 [39]. Pep-7 abolishes the effect of E7 on pRb, whereas its interactions with p130 and p107 are not disturbed. We also found that Pep-7 treatment had no significant influence on the RNA levels of HPV16E7 and pRb. Furthermore, the stabilization of pRb with Pep-7 was addressed by half-life study. In this experiment, pRb half-life was expanded significantly by Pep-7, compared with control (Figure 5D). Therefore, it is reasonable to speculate that Pep-7 may rescue the tumor-suppressing function of pRb by releasing it from the HPV E7/pRB complex and preventing its degradation induced by HPV E7.

HPV E7/pRB association abrogates the transcriptional repressor activity of pRB/E2F complexes, resulting in the dysregulated expression of E2F target genes [17]. On the basis of these concepts and our results mentioned above, it seems possible to ascribe Pep-7 function to the restoration of pRb/E2F pathway and the inhibition of E2F dependent genes. In order to confirm this speculation, a series of gene expression tests were performed. First, results of the qRT-PCR assay indicated that Pep-7 treatment led to a decline in the RNA levels of cyclins A, D1, and E1. Next, Western blot results confirmed the expression reduction induced by Pep-7 (Figure. 4). Finally, the luciferase reporter experiment verified that Pep-7 inhibited E2F transcriptional activity. Pep-7 treatment resulted clearly in activitiy down-regulation of cyclin A, E1 and D1 promoters, whereas this suppression was abolished after the E2F-binding sites were mutated in promoters of cyclin A and cyclin E1 (Figure 6C). Like the E2F-binding sites mutation, knocking down pRb by siRNA abolished the effect of Pep-7 on these E2F response promoters, whereas the selected peptide-mediated inhibition of the above promoter activities did not changed with the p107 and p130 knocked-down (Figure 6B). As the efficiencies of knocking down pRb, p107 and p130 are similar to be about 80%, the different influences on Pep-7 function may be due to the fact that p107 and p130 are not involved in the Pep-7 functional mechanism, which is consistent with the Western blot results that Pep-7 had negligible impact on p107 and p130 protein. Although p130 and p107 may be involved in regulating the activities of members of E2F family, this difference is still reasonable as studies have indicated differences in the interactions of the pRb, p107 and p130 with the members of the E2F family [40], [41], [42]. In conclusion, these results support our hypothesis that the selected peptide Pep-7 demolishes the function of HPV16 E7 protein by inducing its degradation, disrupting its association with pRb, and preventing pRb degradation, which restores the pRb/E2F pathway and suppresses cell proliferation consequently.

Moreover, identical conclusion could be drawn out from animal experiments. In agreement with results in cell lines, Pep-7 showed significant anti-tumor efficacy on SiHa tumor xenografts (Figure 7). BrdU incorporation and Immunohistochemistry assay revealed that similar to that in HPV16-positive cells lines, Pep-7 induced cell growth repression, E7 protein decline, as well as pRb protein accumulation in tumor tissues (Figure 7 and Figure 8). qRT-PCR indicated that Pep-7 also reduced cyclins A, D1, and E1 expression. These results indicate that Pep-7 inhibits tumor growth in animals through the mechanism similar to that in HPV16-positive cells.

Previous studies have revealed that LXCXE of HPV E7 binds entirely through the B pocket domain of pRb [43]. For high risk HPV, the LXCXE motif is also required for pRb degradation [44], while the zinc-binding region of the carboxyl-terminal domain of E7 is important for its dimerization and intracellular stabilization [43], [45]. In this study the Pep-7 has the amino acids sequence SACPKGLDWCGG (C&C bridged). We did not find any similarity of this to the sequences of B pocket domain of pRb. This argued that Pep-7 may not interfere with E7/pRb interaction through binding site competition. Considering the results that the peptide could also compete with E7 antibodies for binding to E7 protein *in vitro*, and induce intracellular degradation of E7, we presume that its association with E7 changed the protein's structure which was critical for its function and intracellular stability. However, the precise mechanisms by which Pep-7 interacts with E7 and releases pRb from E7/pRb complex remain to be elucidated.

In summary, we have explored the possibility of developing new therapeutics for cervical cancer by selecting peptides that bind with high affinity and specificity to the E7 oncoprotein of HPV16. Our study indicates that the selected peptide Pep-7 has anti-tumor efficacy both *in vitro* and *in vivo*. The mechanism studies reveal that the anti-tumor effect of Pep-7 is due mainly to its destruction of HPV16E7 protein function by inducing its degradation and disrupting its interaction with pRb, thus reactivating the pRb/E2F pathway. In light of our results, this study may open new perspectives in the treatment of HPV16-positive cancer.

## Materials and Methods

### Cell lines

HPV16-positive SiHa and CaSki cervical carcinoma cells were supplied by China Center for Type Culture Collection (CCTCC). The following cell lines used in this project were from our laboratory: HPV18-positive HeLa cervical carcinoma cells, CNE nasopharyngeal carcinoma cells, HaCat human keratinocyte cells, COS7 (African green monkey SV40-transformed kidney fibroblast cell line), 293T (human kidney epithelial cells), and NIH/3T3 mouse embryonic fibroblast cells. All cells were maintained in a humidified atmosphere of 5% CO_2_, Dulbecco's modified Eagle's medium (DMEM, pH 7.2), supplemented with 10% fetal calf serum (FCS).

### Antibodies and Reagents

Mouse anti-HPV16E7, mouse anti-p107 (SD107), mouse anti-cyclin E1, mouse anti-cyclin A1 and mouse anti-cyclin D1 monoclonal antibodies were supplied by Santa Cruz Biotechnology, Inc. (Santa Cruz, CA, USA). Rabbit monoclonal to pRb antibody was supplied by Abcam Ltd. (Hong Kong, P.R. China). Mouse monoclonal to p130/Rb2 antibody was supplied from NeoMarkers For Lab Vision Corporation (Fremont, CA, USA). pRb Antibody Sampler Kit, including Phospho-pRb (Ser780), Phospho-pRb (Ser795) and Phospho-pRb (Ser807/811) antibodies and pRb (4H1) Mouse antibody, was supplied by Cell Signaling Technology, Inc. (Danvers, MA, USA). HRP-coupled mouse anti-M13 antibody, HRP-conjugated goat anti-mouse IgG and HRP-conjugated goat anti-rabbit IgG were supplied by Amersham Biosciences (Piscataway, NJ, USA) and KPL (Gaithersbug, MD, USA), respectively. Mouse anti-HPV18E7 serum was prepared in our laboratory. TMB substrate kit was obtained from eBioscience Inc. (San Diego, CA, USA). Ni-NTA agarose beads (His-band Resin) and protein G agarose beads were supplied by Amersham Biosciences (Little Chalfont, UK). The Ph.D.-C7C™ phage display peptide library kit was purchased from New England Biolabs Inc. (Cambridge, MA, USA). DMEM, trypsin, and TRIzol reagent were supplied by Invitrogen (Carlsbad, CA, USA). Fetal calf serums (FCS) were purchased from Sijiqing (Hangzhou, P.R. China). 3-(4, 5-dimethylthiazol-2-yl)-2, 5-diphenyl tetrazolium bromide) (MTT) and dimethylsulfoxide (DMSO) were supplied by Sigma Chemical Co., (Aaint Louis, MI, USA). 5-Bromo-2′-deoxyuridine(BrdU) and Monoclonal Anti-BrdU antibody produced in mouse were bought from Sigma-Aldrich, Inc. (Aaint Louis, MO, USA). Other chemicals were of analytical grade and obtained from local commercial resources.

### Plasmids and Reporter Constructs

The primers used in plasmid constructs of this study are listed in Table 1. HPV16E7-pEYFP plasmid was constructed by sub-cloning the HPV16E7 gene into the *Eco*RI and *Bam*HI sites of pEYFP-N1. The HPV16E7 gene was obtained by PCR from HPV16E7- pET28a (+) plasmid, which was maintained by our laboratory, using primers pE7N1f and pE7N1b. The pRb gene was obtained by nest PCR from HeLa cDNA using pRb1, pRb2, pRb3, pRb4 as primers. The PCR product was then inserted into the *Hind*III and *Xho* I sites of pcDNA3.1-His/V5-Topo. pA3-Luc-Cyclin D1 luciferase reporter plasmid containing the cyclin D1 promoter sequence −1745 to +134 was maintained by our laboratory. Both pCCNE1-Luc and pCCNA1-Luc were constructed by nest PCR to amplify fragments of cyclins E1 and A promoter sequences (cyclin E1: +3 to +594; cyclin A: −284 to +254) which include E2F binding sites [46], [47] from HeLa cDNA, respectively. Primers used in pCCNE1-Luc construction were P(CCE1), P(CCE2), P(CCE3), and P(CCE4), while primers P(CCA1), P(CCA2), P(CCA3), and P(CCA4) were used in PCCNA1-Luc construction. The PCR products were then cloned into the *Kpn*1 and *Bgl* II sites of the pGL3-enhancer (Promega, Madison, WI, USA) luciferase reporter plasmid. The double mutants of E2F sites in the pCCNE1, which changed TGTCCCGC at position +7 to TGTCATGC and changed GCGCGCAA at position +497 to GCGATCAA, were performed by site-directed mutagenesis using the megaprimer-PCR method as described previously [48]. Luciferase reporter plasmid pMCCNE1-Luc, was made by sub-cloning of the corresponding PCR fragments into pGL3-enhancer, respectively. Primers P(pGL31),P(CCME1), P(CCE3), P(CCE4) and P(CCME2) were used in the megaprimer-PCR processes. The E2F binding sites muted cyclin A promoter luciferase reporter plasmid, pMCCNA1-Luc, in which the E2F binding sites TTTGGCTC at position +31 and TTTGGGCG at position +165 were changed into TTTGATTC and TTTGGATG, respectively, was generated form pCCNA1-Luc as above, using P(pGL31), P(CCMA1), P(CCA3), P(CCA4), and P(CCMA2) as primers. The mutations were confirmed with DNA sequencing by Shanghai Sangon Biological Engineering Technology and Service Co., Ltd. (Shanghai, P.R. China).

**Table 1:** Primer sequences used in plasmid constructs.

Name	Primer Sequence (5′-3′)	Name	Primer Sequence (5′-3′)
pE7N1f	CGGAATTCCCACCATGCAT GGAGAT ACA CCT ACA	P(CCE1)	ACCCAGGCTGGTCTCGAAC
pE7N1b	CGGGATCCCGTGGTTTCTG AGA ACA GAT GG	P(CCE2)	TTCGCATCCCTGTGGACC
pRb1	TGTAACGGGAGTCGGGAGAG	P(CCE3)	GTGGGTACCGCTGGGATTAAAGGCGTGAG
pRb2	TGTCCACCAAGGTCCTGAGATC	P(CCE4)	CGTAGATCTGATGGGGCTGCTCCGG
pRb3	CCGAAGCTTGCCACCATGCCGCCCAAAACC	P(CCAM1)	CTACGACTATTCTTTGGATGGGTCGGTGCG
pRb4	CCGCTCGAGCGTTTCTCTTCCTTGTTTGAGG	P(CCAM2)	TCTGGGCGTCTTTGATTCGCCACGCTG
P(CCA1)	AGCCAGGAAATAAGAAGAGC	P(pgl31)	CCAACAGTACCGGAATGC
P(CCA2)	CGTCTGCTGCAATGCTAG	P(CCEM3)	CCGCCCGCGATCAAAGGGGGAAGG
P(CCA3)	CAGGGATCCCACTGCTCCCGGGAGTGG	P(CCEM4)	GGTGGTACCTAAATGTCATGCTCTGAGCCGGG
P(CCA4)	GTGGGTACCGCTTAGAGTCAGCCTTCGGACAG		

### Protein Expression and Purification

The HPV16 E7 gene was amplified by PCR and cloned into the *Bam*HI/*Eco*RI sites of the pET28a (+) vector (maintained by our lab). The pET28a (+)-HPV16E7 clone was then transformed into *Escherichia coli* BL21. Expression of His-E7 was induced by 1 mmol/L IPTG; the protein was extracted in denaturing conditions according to the Qiagen protocol and its purification carried out by immobilized-metal affinity chromatography with Ni-NTA agarose beads following the manufacturer's instructions (Amersham Biosciences, Little Chalfont, UK).

### Phage Display Screening

HPV16E7 protein [100 µg/ml in 0.1 M NaHCO_3_ (pH 8.6) with 5 mg/ml BSA] was immobilized in 96-well microtiter plates (Corning, NY, USA) and incubated overnight at 4°C. The following day, the wells were blocked by incubation for 2 h at 37°C with 3% BSA and rinsed five times with TBST [Tris·Cl (pH 7.5) 50 mmol/L, NaCl 150 mmol/L, and 0.1% Tween 20]. The plates were subjected to panning for 1 h at room temperature with the phage particles, followed by washing three times for 15 min with TBS [Tris·Cl (pH 7.5) 50 mmol/L, NaCl 150 mmol/L]. Thereafter, the bound phages were eluted for 1 h at room temperature with 1 mg/ml HPV16E7 protein in 0.2 M Glycine-HCl (pH 2.2), followed by neutralization with 1.0 M Tris-HCl (pH 9.1). The recovered phages were amplified in Luria-Bertani plates containing stationary phase ER2537 cells. Amplified phages were screened in additional rounds and then plated to obtain isolated clones.

To characterize the individual clones' binding to HPV16E7, plaques were picked at random, amplified, and tested for HPV16E7 binding ability by ELISA using HRP conjugated anti-M13 antibody (Amersham Biosciences, Piscataway, NJ, USA), followed by TMB substrate coloration (eBioscience, San Diego, CA, USA) for colorimetric evaluation. The absorbance value was measured at 450 nm (A450) on a microplate reader. The PBS was immobilized to the plates as negative control. The clone was identified as positive if the A450 value of the sample well was three times higher than that of the negative control well.

### Analysis of Amino Acid in Phage Clones and Peptide Synthesis

The nucleotide sequences coding for random peptides displayed on the positive-phage coat proteins were determined by Shanghai Sangon Biological Engineering Technology and Service Co., Ltd. (Shanghai, P.R. China). The corresponding peptides were chemically synthesized with or without an N-terminal fluorescein label (Chinese Peptide Company, Hanzhou, P.R. China) for further study. The peptide with the same amount of amino acid, but having random sequences, was synthesized as a negative control peptide. Crude peptides were purified by HPLC and checked by mass spectrometry. Except otherwise noted, peptides were dissolved in DMSO to obtain 10 mmol/L solutions, supplemented with equimolar DTT, and stored at −20°C.

### Competitive ELISA Assay

The specificity and affinity of the positive clones and synthetic peptides were determined by competitive ELISA. 400 ng/well HPV16 E7 protein was coated overnight in microtiter wells at 4°C. The positive clones or peptides at different concentrations mixed with HPV16E7 mAb were incubated with the coated E7 protein in microtiter wells for 3 h. The mixture at the same concentration of the library phage and HPV16E7 mAb was set as negative control. The reaction was revealed by incubation with the HRP-conjugated goat anti-mouse antibody (KPL, Gaithrsbug, MD, USA) as described above. HPV16E7 mAb alone at the same concentration, incubated with the coated E7 protein, gave benchmark absorption. The inhibitory ratio was calculated by the following formula: Inhibitory ratio = 100%−(A450 of mixture well/A450 of mAb alone well)×100%.

### Peptide Internalization and Visualization

The 5-FAM labeled peptides were used to monitor the cell uptake and intracellular location of the peptide. SiHa cells were seeded on the coverslips in 12-well plates (1×10^5^/well) and cultured overnight. The DMEM was replaced with a fresh medium, supplemented with 80 µM 5-FAM labeled peptide. Cells were washed three times with a PBS buffer after 8 h and 24 h of incubation, respectively, and then observed under a fluorescence microscope Leica DMI 6000B. Images were captured with Leica AF6500 software (Leica Mikrosysteme Vertrieb GmbH, Wetzlar, Germany). The percentage of cells up-taking F-pep was analyzed by flow cytometry sorting.

### Cell Viability Assay

Cell viability was determined with an MTT assay. Briefly, the cells were seeded into 96-well plates at a density of 4–5×10^3^ cells/well. The cells were allowed to recover for 24 h before the medium was replaced with DMEM supplemented with peptides at different concentrations. The solvent of the peptides was set as the control. The peptide-supplemented medium was replaced daily and the respective concentrations were maintained. The cells were subjected to an MTT cell proliferation assay at different time points. The plates were incubated at 37°C for 3 h with the addition of 20 µl/well MTT (5 mg/ml). Untransformed MTT in the solution was removed by aspiration. The formazan product was dissolved in DMSO, and plates were shaken vigorously at room temperature for 10–15 min to ensure complete solubilization. The optical density of the formazan solutions was measured by microplate reader at 540 nm.

### Clonogenic Assay

Cells were seeded in DMEM at 1×10^3^ cells/well density in 12-well plates and allowed to attach to the substrate overnight. Thereafter, cells were treated with 0 µM, 16 µM, 32 µM, 80 µM, and 160 µM peptide in DMEM, and the medium was changed every three days. The plates were observed daily for colony formation. After 8 (for HeLa cells) or 14 (for SiHa and CaSki cells) days of treatment, the cultures were washed three times with PBS and stained with crystal violet in ethanol for 15 min. The colonies were then counted and photographed with the Alphalmager® EP image acquisition system (Alpha Innotech, Santa Clara, CA, USA).

### Flow Cytometry

Cell cycle distribution was determined by flow cytometry DNA analysis. SiHa cells (2×10^5^/well) were allowed to attach to the substrate overnight and then treated with or without 32 µM and 80 µM Pep-7 in full-culture medium. After 24 h, cells were harvested and counterstained with PI (Invitrogen, Carlsbad, CA, USA) for flow cytometry according to the manufacturer's instructions. Briefly, cells were trypsinized and resuspended in 1 ml PBS. Cell suspension was dropped slowly into 4 ml anhydrous ethanol pre-cooled at −20°C and mixed. After standing at −20°C for more than 15 min, the ethanol was removed by centrifugation, after which 5 ml PBS was added and mixed, and cells were re-hydrated at room temperature for 15 min. PBS was then removed by centrifugation. Cells were stained with 2 µg/ml propidium iodide in 1 ml dilution buffer (100 mmol/L Tris, pH 7.4, 150 mmol/L NaCl, 1 mmol/L CaCl_2_, 0.5 mmol/L MgCl_2_, 0.1% Nonidet P-40, 100 µg/ml RNAase) at room temperature for 15 min and subjected to flow cytometry analysis. Analysis was performed on an Epics-XL flow cytometer (Beckman Coulter, Inc. CA, USA) with Modfit 3.0 software (Verity Software House, Topsham, ME, USA).

### Western Blot

Western blotting was carried out as described previously [49], [50]. The cells were seeded into 6-well plates, allowed to attach to the substrate overnight, and then treated with or without 80 µM Pep-7. After another 48 h of treatment, the cells were lysed in lysis buffer (Beyotime Institute of Biotechnology, Jiangshu, China) containing PMSF (Sigma Chemical Co., St. Louis, MI, USA) for 30 min at 4°C. The lysate was then centrifuged for 20 min at 13,000 rpm and 4°C. The proteins were then separated through SDS-PAGE and transferred onto the PVDF membrane (Immobion®-P Transfer Membrane, Millipore Corp., Billerica, MA, USA). Membranes were blocked in a Tris-buffered saline with 0.1% Tween-20 (TBS-T) solution with 5% nonfat dry milk and incubated overnight with primary antibodies at 4°C. The immunoreactive signals were detected by HRP-conjugated secondary antibodies (KPL, Gaithersburg, MD, USA) followed by SuperSignal® West Pico Chemiluminescent Substrate (Thermo Scientific, Milford, MA, USA.). Anti-β-actin antibodies were used to control protein loading. For the in *vivo* studies, tumors were harvested, and the cell lysates were prepared and transferred to a clean Eppendorf tube and centrifuged for 30 min at 14,000 rpm. The supernate was probed by western blotting, as described above.

For pull-down analysis, the purified His-HPV E7 protein (200 µg/ml×50 µl) was preincubated with 100 µl His-band Resin (Amersham Biosciences, Little Chalfont, UK) for 4 h at 4°C. The beads were then washed four times with lysis buffer and divided into two equal portions. One half was added with the supernate of the 293T cell lysate with 300 µg total proteins and 10 µl (8 mmol/L) Pep-7, while an equal amount of cell lysate supernate and 10 µl PBS as control was added to the other half. The mixtures were incubated overnight at 4°C under continuous agitation. Beads were subsequently washed four times with a lysis buffer. Bound proteins were eluted by boiling in Laemmli sample buffer and analyzed by Western blot.

For Co-IP, Cos-7 cells seeded in 100 mm plates were co-transfected with HPV16E7-pEYFP-N1 and pRb-pcDNA3.1 using the PEI reagent (Sigma-aldich Chemie GmbH, Steinheim, Germany) described previously [51]. Cells were treated with or without 80 µM Pep-7 24 h post-transfection, and lysed for 30 min in lysis buffer 48 h post-transfection. Lysate supernate prepared as above were incubated with 10 µl rabbit anti-pRb antibody or mouse anti-flag (negative control) antibody and washed protein G agarose beads (Amersham Biosciences, Little Chalfont, UK) overnight at 4°C under continuous agitation. Beads were subsequently washed four times with the lysis buffer. Bound proteins were eluted by boiling in a Laemmli sample buffer and subjected to western blot assay.

### Half-life measurement

SiHa cells seeded in 6-well plates were treated without or with 80 µM Pep-7 for 24 h. The cells were then treated with 50 µg/ml cycloheximide for 0 to 12 h. The cell lysates collected at different time points were subjected to Western blotting assay as above. Band intensity was used to determine the proteins half-life [52].

### siRNA Synthesis and Cell Transfection

The siRNAs were synthesized by Shanghai Gene Pharma Co., Ltd. (Shanghai, P.R. China), as follows, E7 siRNA (target sequence: GCTTCGGTTGTGCGT) [53], sense: 5′-GCUUCGGUUGUGCGUACAA dTdT-3′, antisense: 5′-UUGUACGCACAACCGAAGC dTdT-3′, pRb-1, target sequence: 5′-AAGTTTCATCTGTGGATGGAG-3′; pRb- 2, target sequence: 5′-AATGGTTCACCTCGAACACCC-3′; p107-1, sense: 5′-GCCGGUUACAGAGUAUUGUTT-3′; antisense: 5′-ACAAUACUCUGUAACCGGCTT-3′; p107-2, sense: 5′-GCCCUUCUAAGAGUUUGAATT-3′; antisense: 5′-UUCAAACUCUUAGAAGGGCTT-3′; p130-1, sense: 5′-GGACUUAGUUUAUGGAAAUTT-3′; antisense: 5′-AUUUCCAUAAACUAAGUCCTT-3′; p130-2, sense: 5′-GCCCUUCUAAGAGUUUGAATT-3′; antisense: 5′-AUUCUCAACAAAUAGCCGCTT-3′; negative control sense: 5′-UUCUCCGAACGUGUCACGUTT-3′, antisense: 5′-ACGUGACACGUUCGGAGAATT-3′ [54].

The cells were seeded in 12-well plate at a density of 3×10^5^ cells/well. The following day, cells were transfected with SiRNA and luciferase reporter plasmids or siRNA alone, respectively, using LipofectAMINE 2000 as described in the manufacturer's protocol (Invitrogen, Carlsbad, CA, USA).

### Luciferase Gene Reporter Assay

The reporter assays were performed using Bright-Glo™ Luciferase Assay System (Promega, Madison, WI, USA) according to the manufacturer's instructions. Briefly, cells were plated in 12-well plates at a density of 1.5×10^5^ cells/well. The following day, cells were transfected with reporter constructs. After 24 h, cells were pre-treated with or without 80 µM Pep-7 for another 24 h. Thereafter, cell lysates were collected for reporter assays. The luciferase reporter activity of each sample was normalized against the corresponding protein content prior to calculation of the fold induction value relative to the controls. Each sample was determined in triplicate. The results represent means ± SE from three experiment runs.

### RNA Extraction and qRT–PCR

Total RNA was extracted from the cultured cells using TRIzol reagent (Invitrogen, Carlsbad, CA,USA) according to the manufacturer's instructions. qRT-PCR was used to confirm the expression levels of mRNAs. Reverse transcription and qPCR were performed according to the protocol of Reverse Transcriptase System™ and SYBR® premix Ex Taq™ (Perfect Real Time) (TaKaRa, Dalian, P.R. China) on the ABI 7300 Real Time PCR System (Applied Biosystems, Foster City, CA, USA) supplied with analytical software. β-actin mRNA levels were used for normalization. Each sample was determined in triplicate. The results represent means ± SE from three experiment runs. The primers used for real-time PCR [55] are listed in Table 2.

**Table 2:** Primer sequences for real-time PCR.

Cyclin E1 For	5′-GCAGCGAGCAGGAGACAGA-3′	PRb For	5′-TCTACCTCCCTTGCCCTGTTT-3′
Cyclin E1 Rev	5′-GCTGCTTCCACACCACTGTCTT-3′	PRb Rev	5′-CAGAAGGCGTGCACAGAGTGT-3′
Cyclin A For	5′-GTTTCCCCAATGCTGGTTGA-3′	16E7 For	5′ -CG GAATTCCCACCATG CAT GGA GAT ACA CCT ACA-3′
Cyclin ARev	5′-AACCAAAATCCGTTGCTTCCT-3′	16E7Rev	5′-CG GGATCCCGTGG TTT CTG AGA ACA GAT GG-3′
Cyclin D1For	5′-TGTTACTTGTAGCGGCCTGTTG-3′	GAPDH For	5′-CTCATGACCACAGTCCATGCC-3′
Cyclin D1Rev	5′-CCGGAGACTCAGAGCAAATCC-3′	GAPDH Rev	5′-GCCATCCACAGTCTTCTGGGT-3′,

### Mice Maintenance and Subcutaneous Tumor Model

Female Balb/C-nu/nu mice were supplied by the Medical Experimental Animal Center of Guangdong Province (Guangdong, P.R. China). The care and use of laboratory animals have been approved by the Tsinghua University Animal Care and Use Committee, complying with the rules of *REGULATIONS FOR THE ADMINISTRATION OF AFFAIRS CONCERNING EX-PERIMENTAL ANIMALS* (Approved by the State Council of P.R. China)

A subcutaneous tumor model of nude mice was constructed. In brief, six-week-old female nude mice (18±2 g) were subcutaneously inoculated with 1.5×10^7^ SiHa cells. When the tumor size reached about 100 mm^2^, the mice were randomly divided into four groups with 5 mice each, namely, control group, low-dose group, middle-dose group, and high-dose group. The groups were treated once every other day for 16 days, with intravenous injections of PBS or Pep-7 at doses of 400 µg/mouse and 800 µg/mouse, respectively. The mice were raised for another 3 weeks. Tumor sizes were measured with a caliper every three days and tumor volume was calculated using “volume = length×width^2^/2.” At the end of the experiment, all mice were sacrificed and the weights of the detached tumors were measured. Tumor growth inhibition ratio was represented as the tumor weight relative to the control, calculated using the formula, “Relative tumor weight (% of control) = average tumor weight of treatment group/average tumor weight of control group×100%.”

### Immunohistochemistry and H/E Staining

Three or two mice bearing tumor of each group were prelabeled with intra-peritoneal injection of BrdU (100 mg/kg, Sigma-Aldrich, Inc., Aaint Louis, MO, USA) 15 hours before sacrificed. Tumor specimens were then prepared as formalin-fixed, paraffin-embedded sections for hematoxylin-eosin staining, BrdU and pRb immunohistochemical staining as previously described [56], [57]. In brief, immunostaining was performed on formalin-fixed, paraffin-embedded sections using the anti-pRb monoclonal and anti- BrdU monoclonal antibodies, respectively. Before the application of the primary antibodies, an antigen retrieval technique was performed. For pRb assay, the deparaffinized and rehydrated slides were placed in 10 mmol/L citrate buffer (pH 6.0) and heated in a microwave oven for 15 min at 700 W. H_2_O_2_ was used to inhibit endogenous alkaline phosphatase. For BrdU incorporation test, sections were pretreated for 20 min with 0.1% trypsin in PBS at 37°C following the 30 min incubation in 2 N HCl at 37°C for DNA denaturation. After blocked, the slides were then incubated with anti-pRb or anti-BrdU monoclonal antibodies overnight at 4°C under dilutions of 1∶100 and 1∶500, respectively. The antibodies were detected by means of the Strept Avidin-Biotin Complex (SABC) method (Wuhan Boster Biological Technology, Ltd., Wuhan, P.R. China.) according to the manufacturer's instructions. Stained slides were examined under a Nikon ECLIPSE TE2000-U microscope (Nikon Corporation, Tokyo, Japan) and images were collected and analyzed with Pixera Penguin 600CL DiRactor™ (Pixera Corporation, Los Gatos, CA, USA). BrdU or pRb-positive cells were counted in each section. The total cell number was determined by nuclear staining with hematoxylin. At least 1000 total cells for each case were counted.

### Statistics and Data Analysis

All cell culture-based experiments were repeated at least three times unless otherwise indicated. Images of colony formation, cell cycle distribution, cell apoptosis, Western blot, and immunostaining results from representative experiments are presented. The figures were created using Adobe Photoshop® CS graphics program. All data were analyzed by a paired t-test using SPSS 11.0 software. Differences were considered statistically significant at *P*<0.02.
